# Toward a framework for psychedelic clinical trials in adolescents

**DOI:** 10.1186/s13034-026-01064-x

**Published:** 2026-03-10

**Authors:** Christine K. Moore, Henry J. Riordan, Rolana Avrumson, Philippe Auby

**Affiliations:** 1 Worldwide Clinical Trials, Durham, NC USA; 2 CoBTeK (Cognition-Behaviour-Technology) Lab, Côte d’Azur University, Nice, France; 3 Department of Child and Adolescent Psychiatry, University, Children’s Hospitals of NICE CHU-Lenval, Nice, France

**Keywords:** Adolescents, Psychedelics, Clinical Trials, Mental Health, Ethics

## Abstract

Recent clinical trials conducted by both industry and academic institutions have demonstrated the therapeutic potential of psychedelics for a variety of mental health conditions in adults. Considering the neurodevelopmental aspects of many of these disorders and the widespread occurrence of these conditions among younger individuals, there is increasing interest in exploring the potential for psychedelic therapies in the adolescent population. Regulatory agencies globally are also expected to require clinical trials in younger populations once such products are approved for adults. This paper details some key considerations for the design and conduct of such trials along with safeguards and best practices tailored to adolescents, building upon established methodologies from adult trials and emphasizing the importance of increased psychological support and rigorous trial oversight. Our goal is to provide a framework that ensures that future clinical trials involving the adolescent population are performed safely, ethically, and in accordance with the highest scientific and regulatory requirements, and to foster a patient-centric dialog within the regulatory and scientific community.

## Background

Recent industry-sponsored clinical trials have established the therapeutic potential of classic serotonergic psychedelics, including psilocybin, LSD (lysergic acid diethylamide), 5-MeO-DMT (5-methoxy-N, N-dimethyltryptamine) and the entactogen MDMA^1^. (3,4-methylenedioxymethamphetamine) for treating a variety of mental health conditions such as treatment-resistant depression and posttraumatic stress disorder (PTSD) in adults [1–4], even in those who have failed multiple prior treatments. Numerous additional studies are underway in generalized anxiety disorder, substance use disorders, and eating disorders, particularly anorexia nervosa. These psychiatric conditions are also frequently seen in adolescents. According to the World Health Organization (WHO), an estimated 1 in 7 children and adolescents aged 10 to 19 are affected by mental health conditions, with anxiety and depression among the most common diagnoses [5]. In the United States, data from the Centers for Disease Control (CDC) suggest that suicide is now the second leading cause of death among adolescents aged 12 to 18 [6]. Eating disorders are a major concern, particularly anorexia nervosa, remaining the most lethal mental disorder among adolescents.

Despite the Food & Drug Administration’s (FDA) rejection of Lycos’s MDMA-assisted therapy for PTSD in August 2024, interest in psychedelic drug development remains high. As of late 2025, more than 30 companies are developing psychedelics [7]; five of those companies have received breakthrough therapy designation by the FDA and have progressed to late stage trials. As such, the first psychedelic drug approval in the United States for a mental health condition in adults could occur as early as 2026 [8].

Given the prevalence of mental health conditions in younger age groups, a pediatric study plan in the U.S. or Paediatric Investigation Plan (PIP) in Europe will likely be required for newly approved psychedelic drugs. In the U.S., the Pediatric Research Equity Act (PREA) of 2003 [9] was established to ensure that new drugs are appropriately studied for safe and effective use in pediatric populations and grants the FDA the authority to mandate pediatric studies for certain drugs including New Drug Applications (NDAs). In Europe, Paediatric Investigation Plans (PIPs) are mandatory for all new marketing authorization applications (MAAs), even if the product is intended only for adults [10]. These requirements are typically only waived if the condition does not occur in children or if associated studies would not be feasible, as was the case with the FDA approval of Spravato^®^ (esketamine nasal spray) for treatment-resistant depression in 2019. While not considered a psychedelic, the FDA waived the requirement for pediatric studies with Spravato because they deemed studies to be impossible or highly impractical given the low prevalence of treatment-resistant depression in the pediatric population. Spravato was later approved for adults with major depressive disorder (MDD) and acute suicidal ideation or behavior, and in this case the FDA did require pediatric studies in 9 to 17-year-olds with MDD [11]. When approved in Europe, the European Medicines Agency (EMA) waived these requirements for studies in those aged 7 to 12 years on the grounds that the product was likely to be unsafe, requiring studies only in patients 12 to under 18 with MDD at imminent risk for suicide [12]. “Adolescents” are defined here as individuals under the age of 18 to correspond to U.S. and European Union regulatory guidance on the legal age for the ability to provide consent, although psychedelic treatment has not yet been suggested for adolescents under the age of 16 [9, 13].

## Added complexity of psychedelic research in adolescents

There has been growing discussion on whether psychedelic use would be appropriate for pediatric or adolescent populations. Some reviewers have argued that the same ethical logic supporting adult psychedelic trials should support adolescent-appropriate research, especially for high-risk groups with inadequate treatment options [14]. Treatment-resistant depression in adolescents, for example, is strongly associated with not only suicidality, but also carries additional developmental consequences such as altered academic trajectories and higher risk for substance abuse during critical maturational periods [15]. The only existing treatment option for anorexia nervosa, which affects multiple body systems and is among the most lethal psychiatric conditions, is psychotherapy, which has limited efficacy and high rates of relapse [16]. Further, off-label use of pharmacological treatments for anorexia is not evidence-based [17]. Unfortunately, evidence from controlled clinical trials on the safety and effectiveness of psychedelics in those under 18 years of age is extremely scarce, and the few existing studies rely on small sample sizes, less rigorous or dated trial methodologies, or the inclusion of participants with narrow diagnostic profiles. One recent observational study involving older adolescents and young adults (16 to 24 year olds) found that psychedelic use led to significant improvements in psychological well-being similar to the effects observed in adults; however, drug-induced ego dissolution, which is usually associated with positive psychological changes in adults, was less advantageous. Moreover, adolescents reported higher rates of drug-related challenging experiences, including significantly greater paranoia, fear, and persistent perceptual disturbances. These findings suggest a potentially elevated risk associated with psychedelic use in individuals under 25 years of age. The authors concluded that although adolescents may derive psychological benefits from psychedelic experiences comparable to those observed in adults, they are more likely to encounter greater challenges during use irrespective of context, dosage, or prior exposure, and exhibit an increased propensity for developing hallucinogen persisting perception disorder (HPPD)-like symptoms following psychedelic administration [18].

Other potential risks of psychedelic use unique to younger populations have been voiced elsewhere and include possible interference with cognitive, emotional, and personality maturation, and ego dissolution at a time when the sense of self is not yet developed [19–22]. Adolescence is a critical period of ongoing neurodevelopment characterized by continued maturation of prefrontal cortical regions, frontolimbic circuitry, and large scale brain networks involved in emotion regulation, impulse control, and self-referential processing [23] [24]. Classic serotonergic psychedelics acutely modulate overlapping neural networks, including the default mode network/internally oriented mental activity, and trigger neuroplastic changes in the adult brain [25]. Although elevated neuroplasticity during adolescence may facilitate therapeutic learning, it may also heighten susceptibility to maladaptive psychological effects when experiences are destabilizing or poorly integrated. The convergence of psychedelic-associated neuroplasticity with ongoing brain maturation therefore represents both a potential therapeutic opportunity and a potential developmental risk, highlighting the importance of conservative dosing strategies, careful participant selection, and extended developmental follow-up in adolescent trials. The present review focuses on outlining safeguards and recommends best practices for conducting psychedelic trials in adolescents in anticipation of regulatory mandates and public interest, drawing from established clinical trial methodologies in adult populations. Recommendations are detailed below and summarized in Table 1.

**Table 1:** Summary of recommendations for clinical trials of psychedelics in adolescents

Domain	Key recommendations
Risk–benefit & participant selection	• Ensure a thorough independent risk/benefit assessment • Include participants with elevated risk in initial trials • Incorporate oversight from an independent Data Monitoring Committee at both a program and study level
Assent & consent	• Use a comprehensive assent process with both participants and family members • Obtain consent from family members/legal guardians and consider the need for independent discussions • Use a flexible, individualized assent/consent process
Choice of control groups & dosing	• Carefully evaluate the need for use of an inactive placebo • Use a conservative starting dose • Incorporate enhanced safety monitoring • Balance the number of planned dose administrations with safety considerations, burden to participate, and management of non-specific treatment effects
Trial duration & follow-up	• Plan for characterization of the durability of response and appropriate intervals between doses • Incorporate longer term follow-up extending beyond the primary endpoint • Include long-term developmental assessments
Entry criteria	• Use age-appropriate structured interviews for confirming diagnoses • Exclude key comorbidities • Establish sufficient severity and stability of symptoms • Ensure clinicians are qualified to administer diagnostic and disease-specific scales and keep them blinded to cardinal entry criteria
Outcome measures	• Use developmentally appropriate scales • Incorporate assessments of treatment expectancy • Formally assess integrity of blinding • Include assessments of the intensity and characteristics of the psychedelic experience, particularly for challenging experiences and ego dissolution
Safety measures	• Monitor physiological effects and adverse events of special interest • Ensure a physician is present or close by • Ensure an action plan is in place for potential emergent suicidality • Incorporate study stopping rules with monitoring by an independent Data Monitoring Committee
Site expertise	• Use child and adolescent psychiatrists as investigators • Use efficacy raters experienced in the therapeutic area in an adolescent population • Have two dosing session monitors who meet requirements specified in regulatory guidance • Separate dosing session monitors from blinded efficacy raters and ensure they are experienced with providing support during psychedelic dosing and with an adolescent population
Set and setting Preparatory sessions	• Foster a comfortable, relaxing environment with safety in mind • Audio and video record dosing sessions • Include two or more preparatory sessions tailored to adolescents • Involve parental or legal guardians in preparatory sessions, with the adolescent and/or alone
After dosing	• Include two or more integration sessions aligned with the intended therapeutic model and consider involving parents/caregivers • Ensure continuity of care when the study concludes

## Current psychedelic treatment models

Psychedelic treatment models vary in their approach, particularly regarding the level of psychological support provided during dosing sessions. In frameworks intended for psychedelic-assisted psychotherapy, such as the Lykos model for MDMA, administering psychedelics facilitates and augments true psychotherapy. Many academic researchers advocate for this psychedelic-assisted psychotherapy model instead of drug-only approaches, citing safety and ethical considerations in addition to increased therapeutic benefit. Regulatory agencies, however, do not oversee psychotherapy practices; therefore, most pharmaceutical developers implement supportive-only models that exclude psychotherapeutic components. Moreover, psychotherapeutic interventions may enhance expectancy biases, and regulatory guidelines recommend providing justification for including psychotherapy, as well as quantifying its contribution to the overall effectiveness of treatment [26]. While it is intuitive that trials involving younger populations would require more intensive support (or psychotherapy), such an approach would require similar and substantiated justification.

## Balance of risk/benefit

Risk–benefit assessment in pediatric research is guided by the principle that any potential risks must be minimized and justified by anticipated benefits, that interventions posing more than minimal risk require a prospect of direct clinical benefit to the participant, and that the relationship of anticipated benefit to risk is at least as favorable as available alternatives [27]. More than minimal risk, as is the case in most drug trials, applies when the intervention or procedure exceeds a threshold defined by the magnitude of harm or discomfort encountered ordinarily in daily life or during routine physical or psychological examinations. These principles may necessitate that initial research involving psychedelics in adolescent populations be carried out with individuals who are treatment resistant, have demonstrated insufficient response to conventional therapies, or present with more severe conditions associated with elevated risk, such as anorexia. Notably, not all Institutional Review Boards and Ethics Committees are knowledgeable about laws, regulations, and guidelines related to pediatric research and may require additional rationale for the inclusion of minors. In addition, it is paramount in the context of adolescent clinical development to ensure a thorough independent risk/benefit assessment by establishing independent Data Monitoring Committees (DMCs), and such oversight should be considered at the program level, rather than solely at the study level.

## Obtaining informed assent

Obtaining informed consent from adults prior to psychedelic administration presents greater challenges compared to conventional treatments because of the unique nature of psychedelic experiences and the inherent difficulties in articulating them. Obtaining informed assent from an adolescent is further complicated, as by definition, assent applies where individuals do not have full capacity to provide consent. Adolescents pose further unique challenges for psychedelic research because of their variability in cognitive and emotional maturity, which affects the ability to understand risks and benefits. A comprehensive assent process should involve both participants and their family members, accompanied by detailed written information that clearly outlines all known and potential risks associated with psychedelic administration. These include possible impacts on perception, cognition, judgment, temporal and spatial awareness, and emotional responses, with particular attention to challenging experiences such as anxiety, fear, or panic that may persist for extended periods, as well as the risk of trauma re-exposure. Additionally, increased vulnerability and suggestibility must be clearly communicated [22, 28, 29]. Family members/legal guardians also need to provide informed consent, and some researchers have recommended that they participate in discussions separate from the adolescent participant so that each can express their perspectives independently [30]. In some cases, consent and ongoing decision-making may involve nontraditional caregiving arrangements (e.g., foster care, group homes, shared guardianship), requiring additional coordination to ensure ethical participation and continuity of oversight. This calls for a flexible, individualized consent process that balances ethical requirements for assent with legal parental consent [31].

## Protocol design

### Choice of control group and dose levels

At least one adequate and well-controlled clinical study is generally required to meet the substantial evidence standard to establish effectiveness in marketing applications, although selecting an appropriate control group in psychedelic research presents unique methodological and ethical challenges regardless of participant age. Traditional placebo-controlled designs are considered problematic because the pronounced subjective effects of psychedelics make blinding difficult if not impossible. This limitation increases the potential for expectancy bias and may lead to an overestimation of treatment effects. Such considerations are particularly salient in adolescent populations, where placebo responses in psychiatric clinical trials are often larger and more variable than those observed in adults. Therefore, “active placebo” controls (e.g., low, subperceptual dose of the same psychedelic or nonpsychedelic agents that mimic some physiological effects) are often used to help mitigate functional unblinding. Given the increased importance of ethical considerations in pediatric populations, administering an inactive placebo without therapeutic benefit may be considered inappropriate, particularly in cases of severe or treatment-resistant conditions. Therefore, initial studies involving younger participants could employ low, middle, and high active doses instead of a true placebo arm, thereby generating dose-response data to inform subsequent placebo-controlled trials if required. The starting active dose assumed to be therapeutic should be conservative, beginning at the lower end of the predicted therapeutic range, with enhanced safety monitoring through an independent DMC. The number of dose administrations, possibility for re-administration, and the number of study visits should be balanced with safety considerations and the participant and family burden required to participate—a minimal number of visits is recommended to both minimize burden and lower the likelihood of nonspecific treatment effects owing to an overly therapeutic environment.

Regulatory agencies have somewhat different opinions on the required trial duration for psychedelic trials and do not address adolescent trials specifically. Because psychiatric conditions such as MDD, PTSD, or anorexia nervosa are chronic, FDA Guidance notes that trial duration should generally be 12 weeks; the EMA proposes 3 to 12 weeks with some flexibility in the timing of the primary endpoint [26, 32]. Characterization of the durability of response should be planned, with consideration of the recommended interdose intervals for maintenance of effect and the safety and efficacy of repeat dosing [26]. In addition to characterizing durability of response, adolescent psychedelic trials should incorporate longer-term follow-up extending beyond the primary endpoint to monitor developmental functioning. Follow-up assessments should evaluate academic performance, social and peer relationships, identity development, emotional regulation, and risk-taking behaviors, as psychedelic experiences may exert delayed or evolving psychological effects. These assessments should involve reports from clinicians, the participants themselves, as well as parents.

### Entry criteria

Regardless of specific psychiatric indication (MDD, PTSD, anorexia nervosa, etc.), certainty of diagnosis as well as certainty of the absence of confounding comorbidities is paramount. While comorbidities are the rule rather than the exception in child and adolescent psychiatry, this can be facilitated by use of structured diagnostic interviews intended for a younger population, such as the Schedule for Affective Disorders and Schizophrenia for School-Aged Children (K-SADS) or Mini-International Neuropsychiatric Interview for Children and Adolescents (MINI-KID). As with adult psychedelic trials, certain comorbidities including bipolar disorder, moderate-to-severe substance use disorder, obsessive-compulsive disorder, autism spectrum disorder, borderline personality disorder, or any current or prior diagnosis of psychotic disorder in the participant or a first degree relative should be excluded, as should any other clinically significant cardiovascular, hepatic, renal or other major concurrent illness that might interfere with interpretation of study results or constitute a health risk for the participant as evaluated by a physician, at least in early trials. Sufficient severity and stability of symptoms should be ensured prior to randomization, rated on a scale appropriate for use in a younger population (e.g., the Children’s Depression Rating Scale [CDRS-R] for trials in depression or the Clinician-Administered PTSD Scale for DSM-5 Child/Adolescent Version [CAPS-CA-5]) to prevent overdiagnosis and inclusion of inappropriate participants, as well as to guard against possible floor effects. It is also helpful for efficacy ratings to be administered by a qualified, trained independent clinician who is blinded to the protocol-required inclusion criteria, located at the investigative site or located centrally, to avoid inflation of baseline ratings for purposes of study entry and subsequent regression to the mean postbaseline for reasons other than treatment.

Typically, psychedelic trials also require washout of other serotonergics, both as a precaution to avoid serotonin syndrome and to avoid potential reduction of psychedelic effects [22]. Mood stabilizers and antipsychotics should be discontinued.

### Outcome measures

The choice of outcome measures should also be developmentally appropriate, as the clinical presentation of mental health conditions differs between adolescents and adults. For example, loss of energy, weight changes, appetite, and insomnia have been found to be more common in adolescents with MDD, whereas anhedonia/loss of interest and concentration difficulties are more common in adults; therefore, not assessing vegetative symptoms for example could lead to imprecise measurement of change [33]. Teenagers with PTSD may exhibit more impulsive, risky, self-destructive, or aggressive behaviors than their adult counterparts [34]. Heightened irritability, mood swings, anger, and nonsuicidal self-injurious behaviors (e.g., self-cutting) can also be more common in adolescents. Furthermore, because adolescents may enter psychedelic trials with expectations shaped by social media, peer narratives, and prevalent portrayals of psychedelic experiences—which can heighten expectancy bias, placebo effects, influence subjective reporting, and further complicate blinding—assessments of treatment expectancy and prior beliefs about psychedelics should be incorporated. As with adult trials, an assessment of blinding integrity is also recommended to evaluate the impact of functional unblinding [26, 32].

For psychedelic-specific scales, the Mystical Experience Questionnaire (MEQ) is commonly used as a patient-reported measure to assess the intensity and characteristics of a psychedelic experience in adult trials and should also be included in adolescent trials. Although the Challenging Experience Questionnaire (CEQ) and Ego-Dissolution Inventory (EDI) are less often used in adults, these scales are particularly relevant for adolescent trials, where such experiences are more common.

Collectively, these considerations underscore the need for enhanced methodological safeguards to preserve the reliability and interpretability of efficacy data in adolescent psychedelic trials. Considering heightened emotional variability and suggestibility, rigorous measures to protect data integrity are warranted, including enhanced rater training, ongoing interrater reliability monitoring, centralized review of key psychometric assessments, and use of blinded independent raters as previously noted. Together, these approaches may help mitigate baseline inflation, regression to the mean, and rater drift, which can otherwise obscure true treatment effects [35].

## Enhanced safety measures

As in adult trials, physiological safety monitoring by qualified, trained staff should include evaluations of cardiovascular effects, respiratory depression, sedation, or impairment during dosing sessions. Additionally, because participants can remain in a vulnerable state for an extended period, a licensed on-call physician able to reach the clinical site within 15 min in the event of a medical emergency is recommended so that participants are not placed at an unreasonable or significant risk of illness or injury [26]. Monitoring adverse events of special interest (AESIs), such as euphoria, dissociative effects, impaired cognition, attention and psychomotor effects, and inappropriate affect, is also required [26] even though these effects are expected with psychedelic dosing. Currently there is no standardized method for defining and capturing AESIs across psychedelic trials.

Over the past two decades, regulatory agencies have also mandated the formal and proactive assessment of treatment-emergent suicidal ideation in psychiatric trials. Establishing a well-defined action plan for managing cases of suicidal ideation, which may include hospitalization, the initiation of alternative treatments, or referral to appropriate services, is therefore important. Furthermore, data alerts should be established and implemented for suicidality measures outlined in study protocols (such as the Columbia-Suicide Severity Rating Scale [C-SSRS]). These data should be subject to active central monitoring and integrated into both individual and study-wide stopping rules overseen by the independent Data Monitoring Committee (DMC).

## Site expertise and set and setting considerations

Given the considerable developmental variability, elevated comorbidity rates, and distinct clinical presentations of mental health conditions in children and adolescents compared to adults [36, 37], psychedelic research involving younger populations should be overseen by child and adolescent psychiatrists serving as principal investigators. Efficacy raters and dosing session monitors should also have specialized training in the therapeutic area specifically in an adolescent population. Staff should also be knowledgeable about the medical and psychological effects of psychedelics to be able to properly report adverse events that occur both during and after the dosing session [26].

Investigative sites should have at least two dosing session monitors who meet the requirements specified in regulatory guidance; at least one should be a healthcare provider with graduate-level professional training and clinical experience in psychotherapy, licensed to practice independently [26]. It is essential that dosing session monitors are different from efficacy raters to preserve blinding to treatment allocation, as session monitors are likely to be functionally unblinded during dosing. Because psychedelics can elicit intense emotional responses or challenging experiences, particularly where there has been exposure to early trauma (and because adolescents are more likely to have difficulty with emotional regulation), dosing session monitors should also have experience working with psychedelics and with adolescents. For additional safety considerations specific to research with psychedelics, refer to Johnson et al., 2008 [38].

A unique aspect of psychedelic trials is the importance placed on “setting.” Dosing rooms should not be overly clinical, but rather foster a comfortable, relaxing environment, and from a safety perspective, perceptual changes and disorientation that can occur should be considered (e.g., location of bathrooms, avoiding furniture with sharp corners, glass objects, etc.) [38]. We strongly recommend audio and video recording dosing sessions, which has become routine in adult psychedelic trials. These recordings may be reviewed by independent evaluators to ensure participant safety during vulnerable states as well as to safeguard dosing session monitors. Regulatory agencies may also request access to these recordings for oversight purposes. 

### Preparatory sessions

In adult psychedelic trials, a minimum of two preparatory sessions is generally required, each lasting approximately 60 to 90 min and ideally conducted in person. These occur after a participant has been deemed eligible and appropriate to participate in the trial, with the purpose to build rapport and trust between the participant and dosing session monitors, to create a safe and supportive environment prior to dosing, and importantly to educate participants on the psychological and physical effects of psychedelics with guidance on how to handle challenging experiences should they occur. Dosing day procedures, ethical boundaries, and safety protocols should also be discussed along with potential adverse experiences and how these will be managed by the study team. Preparatory sessions for adolescents should accomplish all these goals, while also addressing the increased potential for ego-dissolution and challenging experiences. It may also be useful to devote more time and attention to techniques for managing emotions and behaviors during a session in a younger population [18, 21]. Session monitors should be careful that this rapport and trust, however, does not create a situation in which the participant feels obligated to remain in the study [29]. Forsberg and colleagues also advocate for the inclusion of parental or legal guardian caregivers in preparation for dosing, either together with the adolescent, alone, or in combination, for their own education and to clarify their role in the process [29]. In addition to procedural considerations, investigators should also anticipate broader psychosocial effects of psychedelic experiences within the family system. Psychedelic experiences in adolescents may also alter dynamics within the family and caregiving system, including shifts in autonomy, communication patterns, and caregiver expectations. Changes in emotional expression or self-concept may be interpreted differently by caregivers, increasing the risk of misunderstanding or relational strain [29]. Preparatory sessions should therefore explicitly address caregiving context considerations, align expectations, and support adaptive communication to mitigate unintended psychosocial consequences. The ideal number of preparatory sessions and their format, length, and content of parental inclusion are still uncertain, as is whether adolescent assent is needed for parental involvement [29]. Support should adapt to developmental stage and individual family dynamics and care must be taken not to unintentionally reinforce any present family dysfunction. 

### Postdose integration sessions

Like in preparatory sessions, most adult psychedelic trials also include two or more 60 to 90-minute postdose “integration” sessions focused on emotional processing of the experience, regardless of the therapeutic or supportive model used. Discussions are led by the session monitors to explore emotional, cognitive, and sensory experiences during dosing, to address challenging experiences or unresolved feelings l uncovered during the sessions, and to help participants interpret the insights gained. Forsberg and colleagues noted that involving parents or caregivers in integration sessions may facilitate these goals and further support the development of emotion regulation skills; however, the optimal level of involvement remains unclear [29]. In any case, ensuring that adolescent participants have the social and therapeutic support necessary after an intense psychedelic experience (or lack thereof if allocated to subtherapeutic doses or placebo) is considered integral for positive outcomes [39].

The end of a study can feel like an abrupt cessation of therapy for participants, which is especially significant in psychedelic trials and for adolescents. Given the hours spent working closely with session monitors, participants may form strong bonds, making ongoing support after the last visit particularly important. Once the study concludes, researchers should aim to have normal clinical care of participants resumed [32].

## Conclusions

The growing prevalence of mental health conditions in the younger population with accompanying therapeutic interest in psychedelics, will (and we advocate should) result in increased regulatory demand for adolescent psychedelic clinical trials. The above outlines practical considerations for assent considerations, trial design, and the optimal level of expertise in investigative site staff and need for specialized training for efficacy raters and dosing session monitors. We have also highlighted the importance of tailoring set and setting for an adolescent population, with both preparatory and integration sessions tailored to developmental needs. These recommendations aim to maximize participant safety and data integrity while also proactively responding to the unique vulnerabilities and needs of adolescents participating in psychedelic research with the goal of providing a framework that supports ethical, developmentally informed research and prepares the field for future regulatory review and clinical application. Future research should also explore how these compounds interact with the developing brain, including impacts on neural plasticity, emotional regulation, and long-term cognitive outcomes, while establishing age-specific safety, risk, and tolerability profiles. Further development of ethical and regulatory frameworks for psychedelic research involving minors is also needed.

## Data Availability

Not applicable.

## Abbreviations

AESI: Adverse Event of Special Interest
CAPS-CA-5: Clinician-Administered PTSD Scale for DSM-5 Child/Adolescent Version
CDC: Centers for Disease Control
CDRS-R: Children’s Depression Rating Scale
CEQ: Challenging Experience Questionnaire
C-SSRS: Columbia-Suicide Severity Rating Scale
DMC: Data Monitoring Committee
EDI: Ego-Dissolution Inventory
EMA: European Medicines Agency
FDA: Food & Drug Administration
K-SADS: Schedule for Affective Disorders and Schizophrenia for School-Aged Children
LSD: Lysergic acid diethylamide
MDD: Major depressive disorder
MDMA: 3,4-methylenedioxymethamphetamine
MEQ: Mystical Experience Questionnaire
MINI-KID: Mini-International Neuropsychiatric Interview for Children and Adolescents
MMA: Marketing Authorization Application
NDA: New Drug Application
PIP: Paediatric Investigation Plan
PREA: Pediatric Research Equity Act
PTSD: posttraumatic stress disorder
U.S.: United States
WHO: World Health Organizatʹion

## Footnotes

1 While there is no single, universally accepted definition of “psychedelic, we have used the definition adopted by the FDA to include classic psychedelics, typically understood to be 5-HT2 agonists such as psilocybin and lysergic acid diethylamide (LSD), as well as entactogens or empathogens such as methylenedioxymethamphetamine (MDMA). [26] Ketamine / esketamine are considered hallucinogens rather than classic psychedelics
